# Collagen Type I Containing Hybrid Hydrogel Enhances Cardiomyocyte Maturation in a 3D Cardiac Model

**DOI:** 10.3390/polym11040687

**Published:** 2019-04-16

**Authors:** Sam G. Edalat, Yongjun Jang, Jongseong Kim, Yongdoo Park

**Affiliations:** Department of Biomedical Sciences, College of Medicine, Korea University, Seoul 02841, Korea; samilan@korea.ac.kr (S.G.E.); jyj727@korea.ac.kr (Y.J.)

**Keywords:** matrigel-based hydrogel, embryonic stem-cell, cardiomyocyte maturation, collagen type I, TGF-β1, FGF-4

## Abstract

In vitro maturation of cardiomyocytes in 3D is essential for the development of viable cardiac models for therapeutic and developmental studies. The method by which cardiomyocytes undergoes maturation has significant implications for understanding cardiomyocytes biology. The regulation of the extracellular matrix (ECM) by changing the composition and stiffness is quintessential for engineering a suitable environment for cardiomyocytes maturation. In this paper, we demonstrate that collagen type I, a component of the ECM, plays a crucial role in the maturation of cardiomyocytes. To this end, embryonic stem-cell derived cardiomyocytes were incorporated into Matrigel-based hydrogels with varying collagen type I concentrations of 0 mg, 3 mg, and 6 mg. Each hydrogel was analyzed by measuring the degree of stiffness, the expression levels of MLC2v, TBX18, and pre-miR-21, and the size of the hydrogels. It was shown that among the hydrogel variants, the Matrigel-based hydrogel with 3 mg of collagen type I facilitates cardiomyocyte maturation by increasing MLC2v expression. The treatment of transforming growth factor β1 (TGF-β1) or fibroblast growth factor 4 (FGF-4) on the hydrogels further enhanced the MLC2v expression and thereby cardiomyocyte maturation.

## 1. Introduction

Engineered cardiac models are highly beneficial for understanding the physiology and pathology of the heart by enabling accurate investigation of cellular functions in the laboratory environment. Such models facilitate the development of novel drugs for cardiac disease. Drug discovery and screening are costly and time-consuming processes that are not always successful due to failures in pre-clinical or clinical stages. The potential toxicity of drugs [1] and the differences between the physiology of the human heart and experimental animals [2] are barriers for predicting the outcomes of cardiac-specific drug screening. For this reason, the development of in vitro cardiac models could accelerate the discovery and development of drugs by providing a reliable platform for pre-clinical testing [3].

Recent developments in stem cell technologies have enabled the use of human pluripotent stem cell (hPSC)-derived cardiomyocytes [4] in the development of models that would represent the native human heart tissue. Despite tremendous progress in differentiating cardiomyocytes from pluripotent sources, the in vitro maturation of these cells remains a challenging task. So far, various strategies, including the physical stimulation of cardiomyocytes [5], electrical stimulation [6] and treatment with soluble factors [7] have been used to achieve cardiomyocyte maturation [8]. Often, using a combination of these strategies has shown to improve the cardiomyocyte maturation [9]. For instance, Biowire, a hybrid platform that combines electrical simulation and extracellular matrix elements was particularly effective in achieving maturation in hPSC-derived cardiac tissues [10].

Although the two-dimensional (2D) culture systems have been useful in elucidating many important aspects of cardiomyocyte maturation, the limited resemblance of these systems to the in vivo conditions seen in the three-dimensional (3D) extracellular matrix (ECM) hampers our understanding of cardiomyocyte development. The maturation of cardiomyocyte in 3D environments exhibited a more natural electrophysiological state compared to the cardiomyocytes grown in 2D [11]. Thus, the development of 3D culturing techniques can advance our understanding of cardiomyocyte maturation by providing the grounds for tissue-like physical and biochemical cell-matrix interactions. 

Depending on the cell type, determining the suitable ECM composition with apt physical properties is of importance for 3D experiments. Matrix composition is thought to play an important role in the regulation of cellular differentiation and proliferation [12,13]. Stem-cell derived cardiomyocytes have been shown to differentiate more effectively on decellularized cardiac 3D ECM compared to 2D [14]. Furthermore, the physical properties of the ECM are thought to impact cellular activities such as the beating of cardiomyocytes [15]. Among the ECM components used for 3D models, collagen type I has seen extensive usage because it is an integral component of the ECM in the myocardium [16] and is thought to be important for the proper function of cardiomyocytes. During the development of the chick embryo, collagen type I localized in the subepicardium of the heart has an important role in cardiomyocyte development [17]. Additionally, it is thought to have a modulatory function in calcium handling and electrical activities of atrial cardiomyocytes, which is critical in the regulation of the electrophysiological function of these cells [18]. 

**Figure 1:**
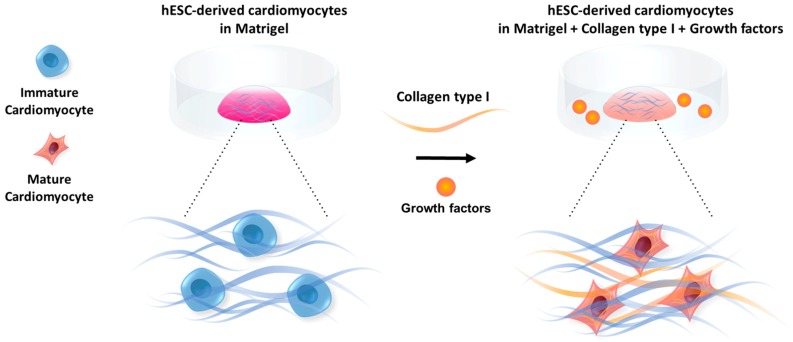
Maturation of cardiomyocytes encapsulated in hydrogels is influenced by the composition and stiffness of the ECM as well as growth factors (GFs).

In this article, we report that the addition of collagen type I as a component of the ECM can enhance the maturation of ventricular cardiomyocytes and that this effect depends on the stiffness of the hydrogel. For this purpose, cardiomyocytes maintained in a variety of hydrogels were cultured for five days and the maturation of the cells was monitored by using the structural analysis and qPCR data. We also showed that treating the cardiomyocytes encapsulated in this hydrogel with transforming growth factor β1 (TGF-β1) or fibroblast growth factor 4 (FGF-4) positively affects maturation (Figure 1).

## 2. Materials and Methods

### 2.1. Cell Culture and Medium Composition

Cardiomyocytes differentiated from H9 embryonic stem-cells were obtained from NEXEL Co., Ltd (Seoul, South Korea). The cells were maintained in 6-well dishes in RPMI medium 1640 (1X) (Thermo Fisher Scientific, Waltham, MA, USA) supplemented with 2% B27 Supplement (50X), minus vitamin A (Thermo Fisher Scientific, Waltham, MA, USA) and 1% Penicillin-Streptomycin (Sigma, St. Louis, Missouri, USA). The medium was replaced every two days. For detachments, the cells were treated with 5 mL of Accutase (Sigma, St. Louis, MO, USA) and incubated at 37 °C for 10 min. The floating cells were centrifuged at 1300 rpm for 3 min. The same medium composition was used for the maintenance of the 3D samples. 

### 2.2. Hydrogel Preparation

Matrigel was made by incubating Matrigel Matrix Growth Factor Reduced (Corning, Corning, NY, USA) for 50 min at 37 °C to reach gelation. For the preparation of 3 mg/mL collagen hydrogel, collagen type I, rat tail (Corning, Corning, NY, USA) and for the 6 mg/mL collagen hydrogel, collagen type I, rat tail, high concentration (Corning, Corning, NY, USA) were mixed with 10x PBS (Thermo Fisher Scientific, Waltham, MA, USA), filtered deionized water and 0.5 NaOH on ice until the pH of the hydrogel reached approximately 7.4. Matrigel based hydrogel solution was mixed with 1 × 10^6^ million cells to form 50 μL of hydrogel for 50 min at 37 °C for 3D culture.

### 2.3. Hydrogel Composition Experiment

For Matrigel with 3 mg/mL collagen type I and Matrigel with 6 mg/mL collagen type I, 0.83 × 10^6^ cells were mixed with 20 μL of Matrigel and 20 μL of 3MG COL hydrogel or 20 μL of 6MG COL hydrogel to form 40 μL drops. The drops were incubated for 50 min at 37 °C to reach gelation. For the Matrigel gel only (MA) samples, 0.83 × 10^6^ of cells were mixed with 40 μL of Matrigel and incubated for 50 min at 37 °C to reach gelation. The images of the hydrogel drops were taken using Eclipse Ti2 microscope (Nikon, Japan). The size of each sample was measured using ImageJ.

### 2.4. Growth Factor Experiment

Cells cultured in the Matrigel-based hydrogel with 3 mg/mL collagen type I were incubated in a medium with growth factors for maturation. The hydrogels with cells were incubated for 50 min at 37 °C to reach gelation. For the FGF-4 (Sigma, St. Louis, MO, USA) treated samples, 50 ng/mL of FGF-4 was added to the medium. For the TGF-β1 treated samples, 10 ng/mL of Recombinant Human TGF-β1 CHO (from derived CHO cells) (Peprotech, Rocky Hill, NJ, USA) was added to the medium. The culture medium was changed every two days and the samples were maintained for 5 days.

### 2.5. RNA Extraction

TRizol Reagent (Invitrogen, Carlsbad, CA, USA) was added to each sample and mixed rigorously using until the gels were dissolved. The samples were then kept in the freezer at −80 °C overnight. After thawing, 200 μL of Chloroform 99.9% (Acros Organics, (Thermo Fisher Scientific, Waltham, MA, USA) was added to each sample. The samples were then mixed for 15 s and following with incubation for 3 min in room temperature. Each sample was inverted 10 times and centrifuged at 15,000 rpm for 15 min at 4 °C. After observing phase separation, the top layer of each sample was transferred to a new tube and 2-Propanol (Sigma, St. Louis, MO, USA) was added in a 1:1 ratio. The samples were inverted 10 times and incubated in the room temperature for 10 min. After 10 min the samples were centrifuged at 15,000 rpm for 10 min at 4 °C. The solution was discarded, and 75% ethanol was added to the samples and centrifuged at 7500 rpm for 5 min at 4 °C. The solution was discarded again, and the pellets were left to dry for 30 min. 30 μL of nuclease free duplex water (Integrated DNA Technologies, Coralville, IA, USA) was added to each sample, and the samples were incubated at 55c °C for ten min. The amount of RNA was measured using Nano drop ND 200-C (Thermo Scientific, Waltham, MA, USA).

### 2.6. cDNA Preparation

cDNA was prepared using PrimScript RT Reagent Kit (Takara, Japan) with Takara PCR Thermal Cycler Dice TP600 (Takara, Japan). The cDNA samples were stored at −20 °C.

### 2.7. Quantitative Polymerase Chain Reaction (qPCR)

The cDNA samples were subjected to qPCR analysis using MyiQ2 (BIO-RAD, Hercules, CA, USA) and IQ SYBR GREEN Supermix (BIO-RAD, Hercules, CA, USA) reagent. As an internal control, the expression of β-actin was evaluated. Gene expression level was estimated as fold changes by comparing the cycle threshold values for the β-actin gene and the mRNAs of interest. List of the primers used for qPCR (Table 1):

### 2.8. Rheometry 

Rheological measurements of the gel formation were performed with rheometer DHR-1 (TA Instruments Ltd., New Castle, DE, USA). The mixtures were carried out in 1 mL solution, on a sandblast parallel plate (diameter 25 mm) under the following conditions: Gelation was monitored for 60 min by observing the viscosity, and elastic modulus measurements were taken at 37 °C in the dynamic oscillatory mode with a constant deformation of 1% and frequency of 1 Hz.

### 2.9. Measurement of Hydrogel Swelling and Degradation

Swelling test was performed on Matrigel, Matrigel with 3 mg/mL collagen type I, Matrigel with 6 mg/mL collagen type I, 3 mg/mL collagen type I (COL3MG) and 6 mg/mL collagen type I (COL6MG). The samples were incubated for 50 min to achieve gelation. After measuring the initial weight, the samples were maintained in deionized water for 24 h and the swollen weight was measured to calculate the swelling ratio. Degradation of the hydrogels by collagenase was measured. Collagenase type I (Worthington Biochemical Corporation, Lakewood, NJ, USA), was added to the DPBS (1x) (Thermo Fisher Scientific, Waltham, MA, USA) to a concentration of 1 U/mL and added to hydrogels maintained in DPBS. The weight loss of the hydrogels was measured at 0, 2, 6 and 18 h. 

### 2.10. Scanning Electron Microscopy of Hydrogel 

The hydrogel samples were fixed with 1% osmium tetroxide for 1 h at 4°C. After standard serial dehydration in ethanol, the samples were freeze-dried using a freezing dryer (Hitachi ES-2030, Japan) and coated with platinum using an ion coater (Eiko IB-5, Tokyo, Japan). The samples were observed using SEM (Hitachi S-4700, Tokyo, Japan).

## 3. Results

### 3.1. Concentration of Collagen Type I in the Hybrid Hydrogels Influences the Hydrogel Morphology and Stiffness

**Figure 2:**
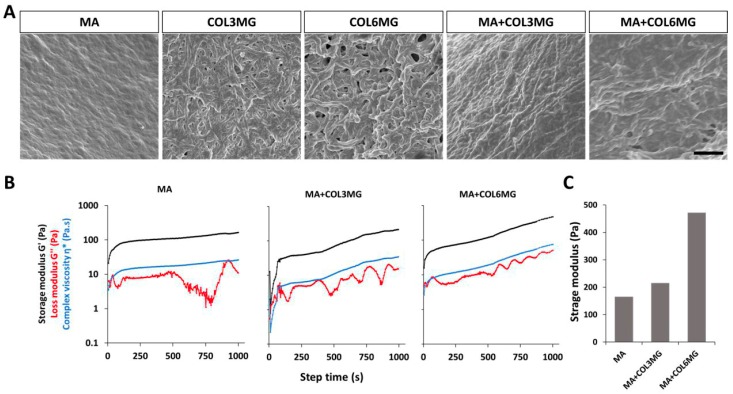
(**A**) SEM images of the hydrogel samples. Scale bar is 1 μm. (**B**) The relationship between the concentration of collagen type I and stiffness of the hydrogels. (**C**) Stiffness was determined using rheometry. Note that MA+COL3MG and MA+COL6MG stand for 3 mg and 6 mg of collagen type I in hydrogel preparations, respectively. Note that SEM and rheometry analysis were performed with n = 1.

We first observed the morphology of the hydrogels using scanning electron microscopy (SEM) (Figure 2). The captured images revealed the differences in the structure of the Matrigel-based hydrogels and the pure collagen samples. There were no pores on the surface of Matrigel whereas collagen gels showed porous structures depending on the concentration of collagen. The higher collagen yielded thicker fibril structures for the hydrogels, and MA+COL3MG and MA+COL6MG revealed denser and thicker fibril structures compared to the MA only samples. In addition, COL6MG showed a more porous structure with thicker fibers than that of COL3MG.

As the ECM stiffness influences many aspects of cellular behavior [19], determining the optimal stiffness [20] is crucial for designing a hydrogel suitable for cardiomyocyte maturation. Since hydrogel stiffness is influenced by the concentration of collagen type I [21], Matrigel-based hydrogels with varying concentrations of collagen type I: (1) Matrigel-only (MA), (2) Matrigel with 3 mg/mL of collagen type I (MA+COL3MG), 3) Matrigel with 6 mg/mL of collagen type I (MA+COL6MG) were prepared and their stiffness were measured using the storage modulus in relation to the gelation time (Figure 2). Each hydrogel shows the different gelation point and elastic response during 2000 s [22]. We took the storage modulus values at 1000 s where each value steadily increased (Figure 2c). The MA+COL6MG hydrogel showed the highest level of stiffness (472 pa), considerably stiffer than both the MA only sample (165 pa) and MA+COL3MG (215 pa).

### 3.2. Effect of Collagen Type I on Swelling and Degradation of the Hydrogels

**Figure 3:**
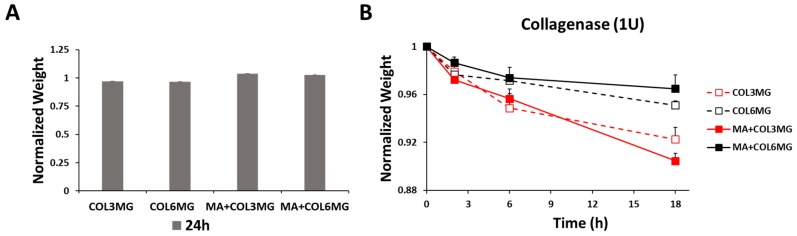
Swelling (**A**) and degradation (**B**) of the hydrogels. Collagenase type I used for the degradation test. Note that the error bars were estimated by the standard deviation of three samples.

To characterize the physicochemical characteristics of hydrogel, the swelling and enzymatic degradation analysis were performed depending on the concentration of the collagen type I and Matrigel (Figure 3). After hydrogel formation, their swelling was monitored for 24 h This suggested a fairly consistent pattern without significant swelling (Figure 3A). To perform the degradation test, 1 units of collagenase type I was used to determine the degradation rates of the hydrogels (Figure 3B). The results showed that the higher concentration of collagen in the hydrogel delayed the degradation time with 1.0 unit of collagenase type I. In addition, Matrigel-based hydrogels followed the same trend with even slower degradation. MA+COL6MG hydrogel showed the slowest degradation rate among the samples in 18 h.

### 3.3. Effect of Collagen Type I on the Structure of the Cardiomyocyte Encapsulated in the Matrigel-Based Hydrogels

**Figure 4:**
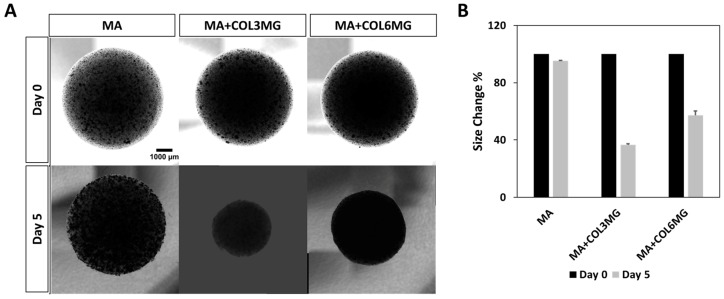
Effects of collagen type I on the shape (**A**) and size (**B**) of cardiomyocyte-containing hybrid hydrogels for 5 days. Note that the error bars were estimated by the standard deviation of two samples.

Given the importance of collagen type I during heart development [23], it was hypothesized that collagen type I could positively influence the maturation of cardiomyocytes in the hybrid hydrogels. The cells were encapsulated in the three hydrogel variants and the changes in the structure of the samples were monitored for 5 days (Figure 4). At the end of the 5 days, MA+COL3MG and MA+COL6MG showed structural differences compared to Matrigel only. The images of the hydrogels showed a more compact for the collagen type I containing hydrogels than that of MA only. There was a 63% reduction in the size for MA+COL3MG variant, a 43% reduction in the size of MA+COL6MG, and only a 5% reduction in the size of MA. This could be explained by the remodeling of the ECM by cells during the 5 days culturing period.

### 3.4. Influence of Collagen Type I on the Expression of Maturation Factors in Cardiomyocytes

**Figure 5:**
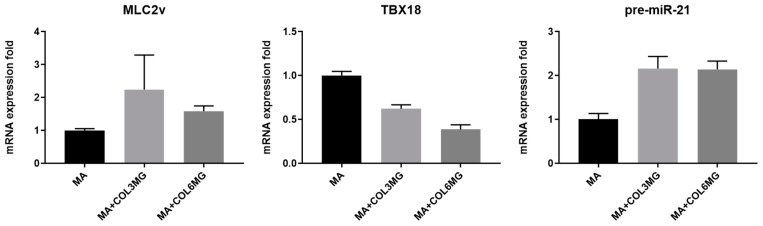
Expression of the cardiomyocyte maturation markers in collagen type I containing hydrogels. Note that the error bars were estimated by the standard deviation of three samples.

To determine the maturation level of the cardiomyocytes maintained in the hydrogels, the expression levels of ventricular myosin light chain-2 (MLC2v) and the T-box transcription factor encoding gene (TBX18) were measured for each variant (Figure 5). MLC2v expression levels are known to increase with maturation of ventricular cardiomyocytes [24,25], whereas TBX18 expression is thought to be high in progenitor cardiomyocytes [26]. Among the hydrogel variants, the expression of MLC2v was upregulated in the collagen type I containing hydrogels. MA+COL3MG and MA+COL6MG showed 2.2-fold and 1.6-fold upregulation in MLC2v expression, respectively. There was also a decrease in the expression of TBX18 in the hybrid hydrogels, with 0.6-fold for MA+COL3MG and 0.4-fold for MA+COL6MG, respectively. The results suggest a higher degree of maturation in collagen type I containing hydrogels, especially in the softer MA+COL3MG variant. We also measured the expression levels of pre-miR-21, a precursor form of the miR-21 microRNA (Figure 4). miR-21 microRNA is known to be involved in the process of hypertrophy [27]. As hypertrophy is thought to be a natural process during the maturation of cardiomyocytes [28], we hypothesized a positive correlation between the state of maturation and the expression levels of pre-miR-21. The results showed 2.1-fold upregulation of pre-miR-21 in both MA+COL3MG and MA+COL6MG.

### 3.5. Effects of FGF-4 and TGF-β1 Treatment on the Structure of Cardiomyocyte-Encapsulating Hybrid Hydrogels

**Figure 6:**
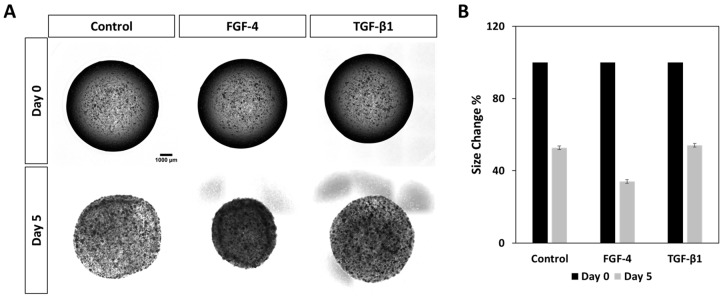
Effects of FGF-4 and TGF-β1 on the shape (**A**) and size (**B**) of cardiomyocyte-containing hydrogels for 5 days. Note that the error bars were estimated by the standard deviation of two samples.

FGFs play a key role in the development of the heart [29], particularly in reprogramming fibroblasts into cardiomyocyte-like cells and maintaining cardiac homeostasis by promoting gap junction formation [30,31]. Another important family of growth factors that are also crucial for the proper development and function of the heart are the TGF-βs [32]. For instance, TGF-β1 is known to be important for Ang II-mediated cardiac hypertrophy [33]. We hypothesized that the treatment of cardiomyocytes encapsulated in MA+COL3MG with FGF-4 and TGF-β1 would have a positive effect on maturation. Cardiomyocytes were treated with 50 ng/mL of FGF-4 or 10 ng/mL of TGF-β1 for 5 days. At the end of this period, the size of the samples was measured (Figure 6). We observed a 47% reduction in size for control, a 46% reduction for TGF-β1 treated samples and a 66% reduction in the size of FGF-4 treated samples. Notably, FGF-4 treated samples showed the highest level of condensation.

### 3.6. Influence of FGF-4 and TGF-β1 Treatment on the Expression of Maturation Factors in Cardiomyocytes

**Figure 7:**
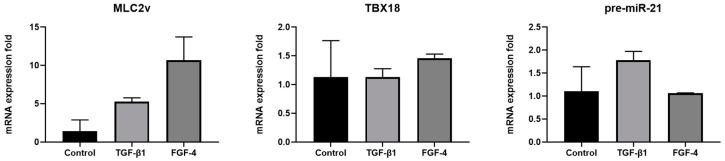
Expression of the cardiomyocyte maturation factors in FGF-4 and TGF-β1 treated hydrogel samples. Note that the error bars are estimated by the standard deviation of three samples.

Samples treated with growth factors enhanced the expression of MLC2v. TGF-β1 treated samples showed 5-fold and FGF-4 treated samples showed a 10-fold increase in the expression of MLC2v. TBX18 expression did not change significantly among the samples. The expression level of pre-miR21 was upregulated by 1.8-fold in TGF-β1 treated samples but remained unchanged in FGF-4 treated sets (Figure 7). These data suggest that FGF-4 and TGF-β1 positively affect maturation, with FGF-4 having the greater effect. TGF-β1 has been shown to increase the expression of miR-21 in cardiac fibroblasts [34]. Thus, we hypothesize that TGF-β1 could be the cause of the higher expression of pre-miR-21 in the TGF-β1 treated sample.

Collagen type I supplemented as a component of the ECM enhances the maturation of ESC-derived cardiomyocytes in 3D environment. This effect is more significant when the concentration of collagen type I and the stiffness of the hydrogel are appropriately adjusted to suit the cardiomyocyte maturation requirements. Our findings suggest that the treatment of the 3D cultured cardiomyocytes with FGF-4 and TGF-β1 enhances the maturation of these cells. Interestingly, we observed a positive correlation between the expression of MLC2v and the reduction in the size of the hydrogel drops. This effect was more significant for the samples treated with FGF-4.

## 4. Discussions

Our finding that the appropriate concentration of collagen type I in the Matrigel-based hydrogels was synergetic for cardiomyocyte maturation in 3D has an important implication for designing the suitable polymer matrix for cardiac tissue engineering and cell transplantation [21,35,36,37]. The stiffness of the ECM is a major determinant in cell-substrate adhesion [38], cell alignment and motility [39]. During the embryonic development of chick hearts, the stiffness of the matrix is a determining factor in the process of cardiac looping [40]. The influence of substrate stiffness can also be observed in cardiomyocytes as well [41,42]. It has been reported that the maturation of neonatal rat ventricular myocytes depends on the substrate stiffness [43]. For cardiomyocytes to undergo maturation in 3D, it is highly required to optimize the stiffness and composition of the hydrogel matrix. Our results showed that for cardiomyocyte maturation, adding 3 mg of collagen type I to Matrigel led to the enhancement of the ECM by tuning the stiffness and composition. 

In this experiment, the reduction in size was accompanied by the contractions of the gel structure, presumably pointing towards an increase in stiffness. It has been suggested that changes in the organization of the ECM may be the reason for the increase in the stiffness of the heart walls after birth [44,45]. These results show a direct correlation between the size and contraction of the hydrogels. We also found that the expression of MLC2v, a maturation marker expressed in cells residing in the ventricular chamber of the heart, is correlated with the size and composition of the hydrogels. 

FGF is thought to be important during the development of the heart [30,46,47]. Despite showing that FGF-4 positively influences the maturation of cardiomyocytes in 3D, the precise mechanism by which it induces maturation remains unknown and the molecular pathways used by FGF-4 to influence maturation are yet to be determined. Our data showed that FGF-4 treatment favored the maturation of the cardiomyocytes accompanied by the contraction of the ECM structure. We hypothesize that FGF-4 treatment caused the remodeling of the ECM by stimulating the fibroblasts in the samples, leading to the contraction of the hydrogels [21].

## 5. Conclusions

In this study, we show the importance of ECM composition and stiffness on the maturation of cardiomyocytes in 3D. Collagen type I is an abundant element of ECM and is seemingly effective for the in vitro maturation of cardiomyocytes. Growth factors including FGF-4 and TGF-β1 have been also shown to be a key player for the maturation of cardiomyocyte. Thus, our results can help develop advanced in vitro systems to study cardiomyocyte maturation and provide the basis of a bio macromolecular based hydrogel composition that can be used for cardiac regeneration, drug screening, and cardiac toxicology.

## Figures and Tables

**Table 1:** Primer information used for qPCR analysis.

Gene	Forward Primer	Reverse Primer	Company
MLC2v	CGGAGAAGAGAAGGACTAGGA	ACAGACAAGGTAGGGACAGA	Integrated DNA Technologies (USA)
TBX18	CTGGATGACCAAGGCCATATTA	ACAGGCTTGATGGGAGAAAG	Integrated DNA Technologies (USA)
pre-miR-21	TGTCGGGTAGCTTATCAGAC	TGTCAGACAGCCCATCGACT	Integrated DNA Technologies (USA)
β-actin	CTTCTACAATGAGCTGCGTGTGGCTC	GTACATGGCTGGGGTGTTGAAGGTC	Integrated DNA Technologies (USA)
